# Distinct roles of Shh and Fgf signaling in regulating cell proliferation during zebrafish pectoral fin development

**DOI:** 10.1186/1471-213X-8-91

**Published:** 2008-09-23

**Authors:** Sergey V Prykhozhij, Carl J Neumann

**Affiliations:** 1 Developmental Biology Unit, European Molecular Biology Laboratory, Meyerhofstrasse 1, Heidelberg, Germany

## Abstract

**Background:**

Cell proliferation in multicellular organisms must be coordinated with pattern formation. The major signaling pathways directing pattern formation in the vertebrate limb are well characterized, and we have therefore chosen this organ to examine the interaction between proliferation and patterning. Two important signals for limb development are members of the Hedgehog (Hh) and Fibroblast Growth Factor (Fgf) families of secreted signaling proteins. Sonic hedgehog (Shh) directs pattern formation along the anterior/posterior axis of the limb, whereas several Fgfs in combination direct pattern formation along the proximal/distal axis of the limb.

**Results:**

We used the genetic and pharmacological amenability of the zebrafish model system to dissect the relative importance of Shh and Fgf signaling in regulating proliferation during development of the pectoral fin buds. In zebrafish mutants disrupting the *shh *gene, proliferation in the pectoral fin buds is initially normal, but later is strongly reduced. Correlating with this reduction, Fgf signaling is normal at early stages, but is later lost in *shh *mutants. Furthermore, pharmacological inhibition of Hh signaling for short periods has little effect on either Fgf signaling, or on expression of G1- and S-phase cell-cycle genes, whereas long periods of inhibition lead to the downregulation of both. In contrast, even short periods of pharmacological inhibition of Fgf signaling lead to strong disruption of proliferation in the fin buds, without affecting Shh signaling. To directly test the ability of Fgf signaling to regulate proliferation in the absence of Shh signaling, we implanted beads soaked with Fgf protein into *shh *mutant fin buds. We find that Fgf-soaked beads rescue proliferation in the pectoral find buds of *shh *mutants, indicating that Fgf signaling is sufficient to direct proliferation in zebrafish fin buds in the absence of Shh.

**Conclusion:**

Previous studies have shown that both Shh and Fgf signaling are crucial for outgrowth of the vertebrate limb. The results presented here show that the role of Shh in this process is indirect, and is mediated by its effect on Fgf signaling. By contrast, the activity of the Fgf pathway affects proliferation directly and independently of its effect on Shh. These results show that Fgf signaling is of primary importance in directing outgrowth of the limb bud, and clarify the role of the Shh-Fgf feedback loop in regulating proliferation.

## Background

During the development of multicellular organisms, pattern formation must be precisely coordinated with proliferation and differentiation. Given that only a relatively small number of signaling pathways are used to direct both pattern formation and cell proliferation during development, it is clear that cell fate specification and cell division are highly context-dependent read-outs of signaling in a given tissue or organ. Activation of a particular signaling pathway, such as the Hedgehog pathway, can stimulate proliferation in one cell type, while activation of the same pathway in another cell type has no effect on proliferation. Moreover, the observation that identical signaling pathways can regulate both pattern formation and cell proliferation provides a mechanism for coordination of these distinct behaviours.

The vertebrate limb is an excellent model system in which to study the interplay between pattern formation and cell proliferation. Limb development is highly amenable to experimental and genetic manipulation in several model organisms, and the main signaling pathways that direct limb development are well characterized (reviewed in [1-3]). Three signaling centers are required for pattern formation and growth in the developing limb bud, two of which we chose to study in this work. One of these is the zone of polarizing activity (ZPA), a small group of cells in the posterior mesenchyme, which controls polarity along the anterior/posterior axis [4]. The secreted signaling protein Sonic hedgehog (Shh) is expressed in the ZPA, and has been shown to mediate the effect of the ZPA during limb development [5-8].

The apical ectodermal ridge (AER) is another major signaling center of the limb bud which runs along its distal margin, and which is the site of expression of several Fgf genes (reviewed in [9]). The AER is required for outgrowth and patterning of the limb along its proximal/distal axis, and can be functionally replaced by FGF-soaked beads in chicken embryos, indicating that Fgf signaling can mediate AER function [10,11]. Furthermore, conditional inactivation of both Fgf4 and Fgf8 in the mouse AER leads to failure of proximal/distal outgrowth [12], thus identifying these members of the Fgf family as the main mediators of AER signaling. Factors from the AER and ZPA form a mutual feedback loop, thereby allowing growth and patterning of the different axes to be coordinated. Thus *fgf-4*, which is expressed in the posterior AER, can be induced in the anterior AER of the chicken limb bud by ectopic Shh protein [13,14]. Furthermore, removal of Shh activity from the zebrafish fin buds leads to loss of *fgf4 *and *fgf8 *expression in the AER [8], and, conversely, removal of Fgf4 and Fgf8 activity from the mouse AER leads to loss of *shh *expression in the ZPA [12], indicating that each signaling pathway is required for the maintenance of the other pathway.

Members of both the Hh and Fgf family of signaling proteins have been shown to function as mitogens in several contexts. Indeed, Fgf1 and Fgf2 were initially identified as mitogenic factors in fibroblast tissue culture, and subsequently, other members of the FGF protein family were found to have a similar activity [15]. Furthermore, Fgf signaling has also been shown to have mitogenic activity *in vivo *during embryonic development. Thus FGF-4 is necessary for proliferation of the inner cell mass during early post-implantation development in the mouse [16], and FGF-8 and FGF-17 are required for proliferation in the mouse dorsal midbrain [17]. Additionally, Fgf signaling promotes proliferation of osteoblasts [18], of lens cells [19], and during hematopoiesis [20].

Like the Fgf family, members of the Hh family function as mitogens in a number of contexts. The Hh signaling pathway has been linked to several cancers, including basal cell carcinoma, pancreatic tumors, and digestive tract tumors, and may be upregulated in as many as 25% of tumors [21-25]. In addition to this oncogenic effect, Hedgehog signaling also directs proliferation during normal development, including in the mouse cerebellum [26], in the *Drosophila *eye [27], in mammalian keratinocyctes [28], and in the mammalian kidney [29]. In several cases Hh signaling has been shown to stimulate cell-cycle progression by causing transcriptional upregulation of D-type and E-type cyclins in target cells [27,30-32]. This transcriptional up-regulation of cell-cycle genes in some instances has been shown to occur as a direct response to promoter binding of members of the GLI family, the zinc-finger transcription factors which transduce Hh signaling to the nucleus [27,32,33].

Since there is clear evidence that both Shh and Fgf signaling are important for outgrowth of the vertebrate limb bud, and since both signaling pathways are known to have a mitogenic effect during development, this raises the question of the relative contribution of Shh and Fgf signaling to regulation of proliferation in the limb bud. This issue is complicated by the feedback loop operating between the two signals, as inhibition of either signaling pathway leads to loss of the other signaling pathway. Laufer and colleagues have previously addressed this issue by removing the AER from chicken wing buds and adding back either FGF4-soaked beads, or *shh*-expressing virus [13,14]. Their results show that Shh alone is insufficient to induce mesodermal proliferation, whereas FGF4 alone is sufficient to do so, leading to them to conclude that the effect of Shh on mesodermal proliferation is indirect, and due to the induction of Fgfs in the AER. However, a recently published study [34] shows that Shh is sufficient to induce *cyclin D1 *expression in the mesoderm of chicken wing buds after AER removal. This observation raises a third possibility: that Shh and Fgf signaling both contribute to the regulation of limb bud proliferation.

To distinguish between these possibilities, we have made use of the genetic, embryological, and pharmacological tools of the zebrafish model system to uncouple the activity of Shh and Fgf signaling in the pectoral fin buds, and to investigate their individual effects on proliferation. In order to categorically uncouple the effect of Fgf signaling from Shh, we implanted FGF4-soaked beads into the limb buds of *shh *mutants. Our data confirm that Fgf signaling is of direct and crucial importance for growth and cell-cycle progression in the limb bud, whereas the effect of Shh on proliferation is indirect, and is mediated via its effect on Fgf expression in the AER.

## Results

### Expression of G1- and S-phase cell-cycle genes in *shh *mutant fin buds is initially normal, but is lost at later stages of development

**Figure 1:**
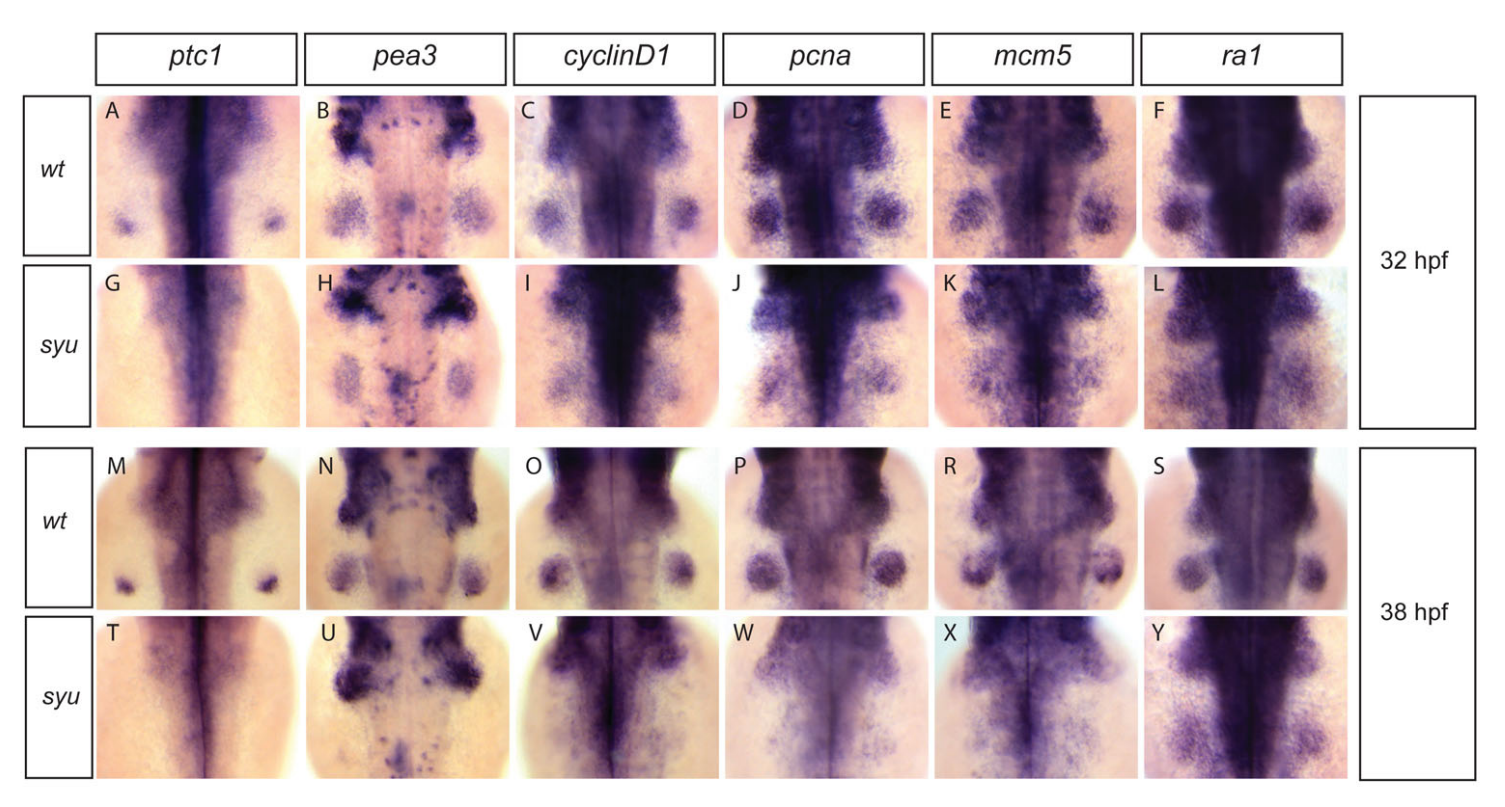
**G1- and S-phase cell-cycle gene expression in fin buds of *sonic-you *mutant correlates with the Fgf signaling status**. Wild-type embryos and *sonic-you *mutant embryos (in which the zebrafish *shh *gene is disrupted) at 32 hpf (A-L) and 38 hpf (M-Y) were analysed for expression of the Shh target *patched1(ptc1) *(A, G, M; T), the Fgf target *pea3 *(B, H, N, U), the cell-cycle genes *cyclinD1*, *pcna*, and *mcm5 *(C-E, I-K, O-R, V-X), and *replication protein A1*(*ra1*) (F, L, S, Y). The Shh target *ptc1 *was expressed in the posterior part of wild-type fin buds at 32 and 38 hpf stages (A, M), but its expresssion was absent in *sonic-you *mutant fin buds (G, T). The Fgf signaling target *pea3 *was expressed at comparable levels in wild-type and *sonic-you *fin buds at 32 hpf stage (B, H). At 38 hpf *pea3 *was still strongly expressed in the wild-type fin buds (N), but almost completely downregulated in the *sonic-you *mutant fin buds (U). *cyclinD1*, *pcna *and *mcm5 *were expressed strongly in both wild-type and *sonic-you *fin buds at 32 hpf stage (C-E, I-K). At 38 hpf these genes were still strongly expressed in the wild-type fin buds (O-R), but downregulated in the *sonic-you *mutant fin buds (V-X). Expression of *ra1 *was similar in both wild-type and *sonic-you *fin buds at 32 and 38 hpf stages.

In order to investigate the role of Shh in regulating cell-cycle progression in the limb bud, we analyzed the expression of cell-cycle genes in the pectoral fin buds of zebrafish *shh *mutants. We focused on *cyclinD1*, *pcna *and *mcm5*, which are generally used as markers of proliferating cells in zebrafish [35,36]. Expression of *cyclinD1 *is necessary for G1-progression and S-phase entry, while *pcna *and *mcm5 *are necessary for DNA replication during S-phase [37]. As a control we analyzed expression of *replication protein A1 *gene (*ra1*), which is expressed constitutively in all cells of the fin bud. We find that at 32 hpf, *cyclinD1*, *pcna*, *mcm5 *and *ra1 *are expressed at indistinguishable levels in wild-type and in *shh *mutant fin buds (Fig. 1C–F, I–L). Since expression of the Shh-target *patched1 *[38] is absent from *shh *mutant fin buds at all stages (Fig. 1A, M), these results indicate that expression of G1- and S-phase cell-cycle genes is independent of Shh at 32hpf. Examination of these cell-cycle genes at 38hpf, however, reveals that *cyclinD1*, *pcna*, and *mcm5 *expression are lost in *shh *mutant fin buds, while *ra1 *remains unaltered (Fig. 1O–R, V–X), suggesting that cell-cycle progression becomes dependent on Shh signaling at later stages. Since the expression of Fgf ligands in the AER depends on Shh activity [1,8], we also tested whether the activity of the Fgf signaling pathway in *shh *mutant fin buds correlates with the observed reduction in cell-cycle gene expression. Using the Fgf-target *pea3 *as a marker for Fgf signaling [39], we find that *pea3 *expression in *shh *mutant pectoral fin buds is identical to wild-type fin buds at 32hpf, but is strongly reduced at 38hpf (Fig. 1B, H, N, U). This result is consistent with the observation that Shh is necessary for maintenance of Fgf expression in the AER, and suggests a correlation between the activity of Fgf signaling and the expression of cell-cycle genes in *shh *mutant fin buds. Taken together, these results show that expression of G1- and S-phase cell-cycle genes is initially normal in *shh *mutant pectoral fin buds, but is later lost, and that this shift correlates with a similar loss of Fgf signaling activity at later stages.

### Loss of expression of G1- and S-phase genes after inhibition of Shh signaling correlates closely with reduction of Fgf signaling

**Figure 2:**
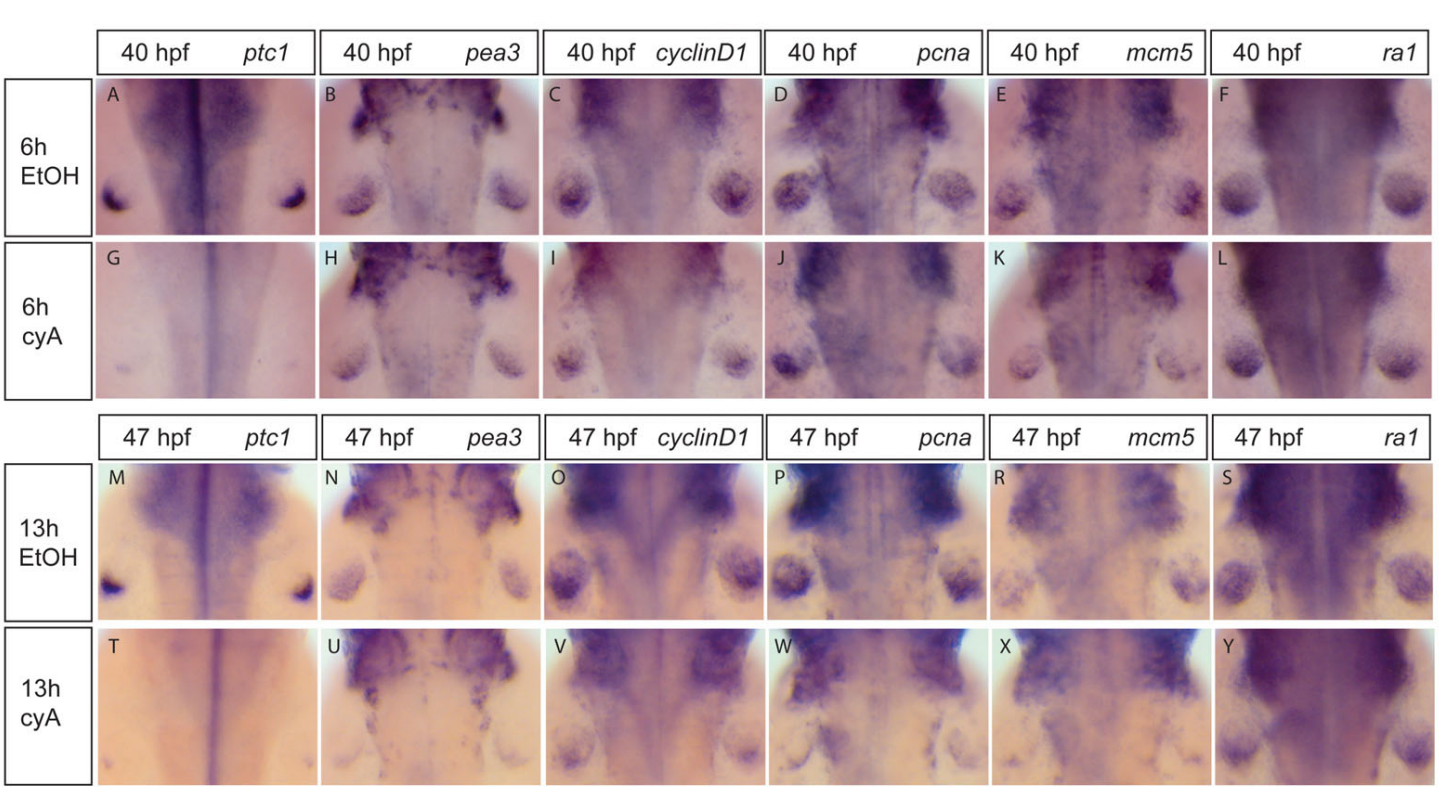
**Expression of G1- and S-phase cell-cycle genes fails to correlate with the status of Shh signaling in fin buds**. Wild-type embryos were treated with 100 μM cyclopamine (cyA) (G-L, T-Y) or with the carrier 0,5% ethanol (EtOH) (A-F, M-S) for 6 hours from 34 to 40 hpf (A-L) or for 13 hours from 34 to 47 hpf (M-Y) and analysed for the expression of *ptc1*, *pea3*, *cyclinD1*, *pcna*, *mcm5 *and *ra1*. Expression of *ptc1 *was nearly completely lost after both 6 hours (A, G) and 13 hours (M, T) inhibition pulses. A 6-hour Hedgehog signaling inhibition led to a small change in *pea3 *expression in fin buds (B, H). Comparably small changes in expression after 6-hour Hedgehog signaling inhibition were observed for *cyclinD1*, *pcna*, *mcm5 *and *ra1 *(C-F, I-L). After 13-hour Hedgehog signaling inhibition, fin bud *pea3 *expression was strongly decreased (N, U). Likewise, expression of *cyclinD1*, *pcna *and *mcm5 *in fin buds was strongly downregulated (O-R, V-X). Expression of *ra1*gene was only mildly affected by 13-hour cyclopamine treatment (S, Y).

Since the loss of G1- and S-phase cell-cycle genes in *shh *mutant fin buds occurs relatively late, and only after Fgf signaling is lost, we decided to use selective inhibition of Hh signaling using the plant alcaloid cyclopamine [40] to determine the time period of inhibition necessary to affect cell-cycle progression. Cyclopamine inhibits the action of Smoothened protein, which transduces the Hh signal after it becomes released from Patched1-mediated inhibition [41]. The use of cyclopamine allows inhibition of Hedgehog signaling for varying periods of time, and thereby temporal control over the signaling inhibition. Our aim was to find a duration of cyclopamine treatment sufficient to inhibit Hedgehog signaling, but leaving Fgf signaling largely unaffected, thereby uncoupling the two pathways from each other. We find that a 6-hour treatment from 34 to 40 hpf with 100 μM cyclopamine is sufficient to inhibit *patched1 *expression almost completely (Fig. 2A, G), but has little effect on expression of the Fgf-target *pea3 *(Fig. 2B, H). Likewise, this treatment has little or no effect on *cyclinD1*, *pcna*, *mcm5 *and *ra1 *expression (Fig. 2C–F, I–L). In contrast, however, a 13-hour cyclopopamine treatment from 34 to 47 hpf leads to loss of both *ptc1 *and *pea3 *expression (Fig. 2M, N, T, U), and also leads to strong reduction of *cyclinD1*, *pcna*, and *mcm5 *expression, but without affecting *ra1 *(Fig. 2O–S, V–Y). These results show that loss of Shh signaling leads to loss of cell-cycle gene expression only after a 13-hour delay, indicating that this is likely to be an indirect effect. Since after this delay period cell-cycle gene expression loss correlates closely with reduction of Fgf signaling in response to Shh inhibition, Fgfs are very good candidates for mediating the effect of Shh on cell-cycle progression in the fin bud.

### Inhibition of Fgf signaling with SU5402 leads to rapid loss of G1- and S-phase cell-cycle genes in the fin bud

**Figure 3:**
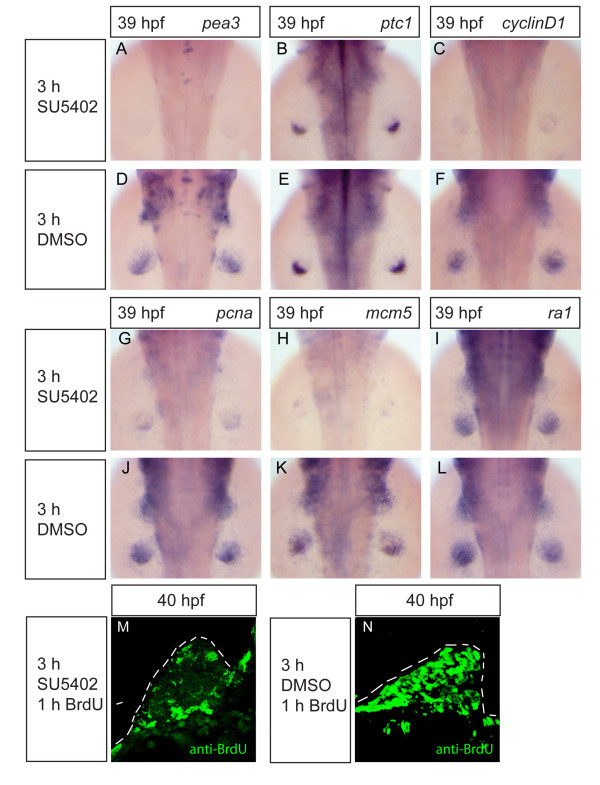
**Inhibition of Fgf signaling causes rapid loss of G1- and S-phase cell-cycle gene expression, and blocks S-phase progression**. Wild-type embryos were treated with 10 μM SU5402 (D-F, J-L, M, N) or the carrier 0,125% DMSO (A-C, G-I, O, P) for 3 hours from 36 to 39 hpf. For BrdU stainings, after 3-hour treatment with either SU5402 or DMSO, embryos were injected with 10 mM BrdU solution into the yolk and incubated for 1 hour in the same solutions before fixation. SU5402 treatment strongly downregulated the expression of *pea3 *FGF signaling target (A, D), but had only a small effect on *ptc1 *expression in fin buds (B, E). SU5402 treatment also caused strong downregulation of *cyclinD1 *(C, F), *pcna *(G, J) and *mcm5 *(H, K). Expression of *ra1 *was not affected by SU5402 treatment (I, L). S-phase progression, as revealed by BrdU labelling, was strongly inhibited after SU5402 treatment, in comparison to control embryos (n = 10, at least 2 fin sections per embryo were analysed) (M, N).

Following our observation that inhibition of Shh signaling strongly affects expression of cell-cycle genes after a 13-hour cyclopamine treatment, we next decided to investigate how rapid the response of the same genes is to inhibition of Fgf signaling. For this purpose, we took advantage of the chemical inhibitor SU5402, which inhibits signaling by Fgf receptors [42], and which has been shown to inhibit Fgf signaling effectively in zebrafish embryos [39]. We find that treatment with 10 μM SU5402 for 3 hours between 36 and 39 hpf leads to nearly complete loss of the Fgf-target *pea3 *(Fig. 3B, E), while expression of the Shh target *ptc1 *is hardly affected (Fig. 3A, D), indicating that, under these conditions, Fgf signaling is blocked whereas Shh signaling is still intact. We find that this 3-hour inhibition of Fgf signaling is sufficient to cause a nearly complete loss of expression of *cyclinD1 *(Fig. 3C, F), *pcna *(Fig. 3G, J) and *mcm5 *(Fig. 3H, K) in fin buds, while *ra1 *(Fig. 3I, L) is unaffected. Interestingly, *cyclinD1*, *pcna*, and *mcm5 *expression are also lost from the branchial arch primordia following this treatment (Fig. 3C, F, G, J, H, K). Consistent with the loss of expression of G1- and S-phase genes after 3 hours of SU5402 treatment, the number of cells labelled with BrdU is also strongly reduced in the fin buds under these conditions (Fig. 3M, N). These results show that the effect of Fgf signaling on cell-cycle progression in the fin buds is much more rapid than the effect of Shh signaling, since there is a severe down-regulation of cell-cycle genes already after 3 hours of inhibition of Fgf signaling.

### Fgf signaling is not generally required for cell-cycle progression in the zebrafish embryo

**Figure 4:**
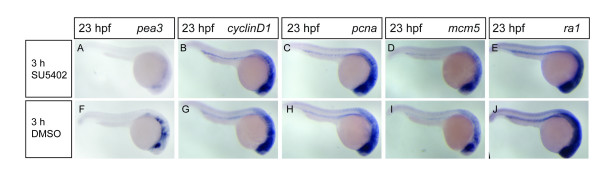
**Fgf signaling inhibition does not cause global downregulation of G1- and S-phase cell-cycle gene expression**. Wild-type embryos were treated with 10 μM SU5402 (A-E) or the carrier 0,125% DMSO (F-J) for 3 hours from 20 to 23 hpf. SU5402 treatment caused a strong downregulation of *pea3 *gene expression (A, F), but expression of *cyclinD1*, *pcna*, *mcm5 *and *ra1 *genes was not changed in SU5402-treated embryos compared to control ones (B-E, G-J).

Since we observed that blockage of Fgf signaling with SU5402 leads to rapid loss of G1- and S-phase gene expression both in the pectoral fin buds and in the branchial arches, we also checked whether Fgf signaling is required for proliferation in other tissues. We therefore performed an inhibitor treatment at 20 hpf, a stage at which many embryonic cells are still proliferative. After 3 hours of treatment with 10 μM of SU5402, expression of *pea3 *is almost completely lost in these embryos, but *cyclinD1*, *pcna*, *mcm5 *and *ra1 *expression is unaltered (Fig. 4A–E, F–J). Furthermore, while SU5402 treatment at 39hpf leads to loss of cell-cycle genes from both the pectoral fin buds and the branchial arches, it has no effect on the same genes expressed in the retina and the optic tectum (data not shown). These results indicate that Fgf signaling is not generally required for proliferation in the whole embryo, but that it instead directs expression of cell-cycle genes specifically in the pectoral fin buds and in the branchial arches.

### Implantation of Fgf4-soaked beads is sufficient to restore expression of G1- and S-phase cell-cycle genes and S-phase progression in *shh *mutant fin buds

**Figure 5:**
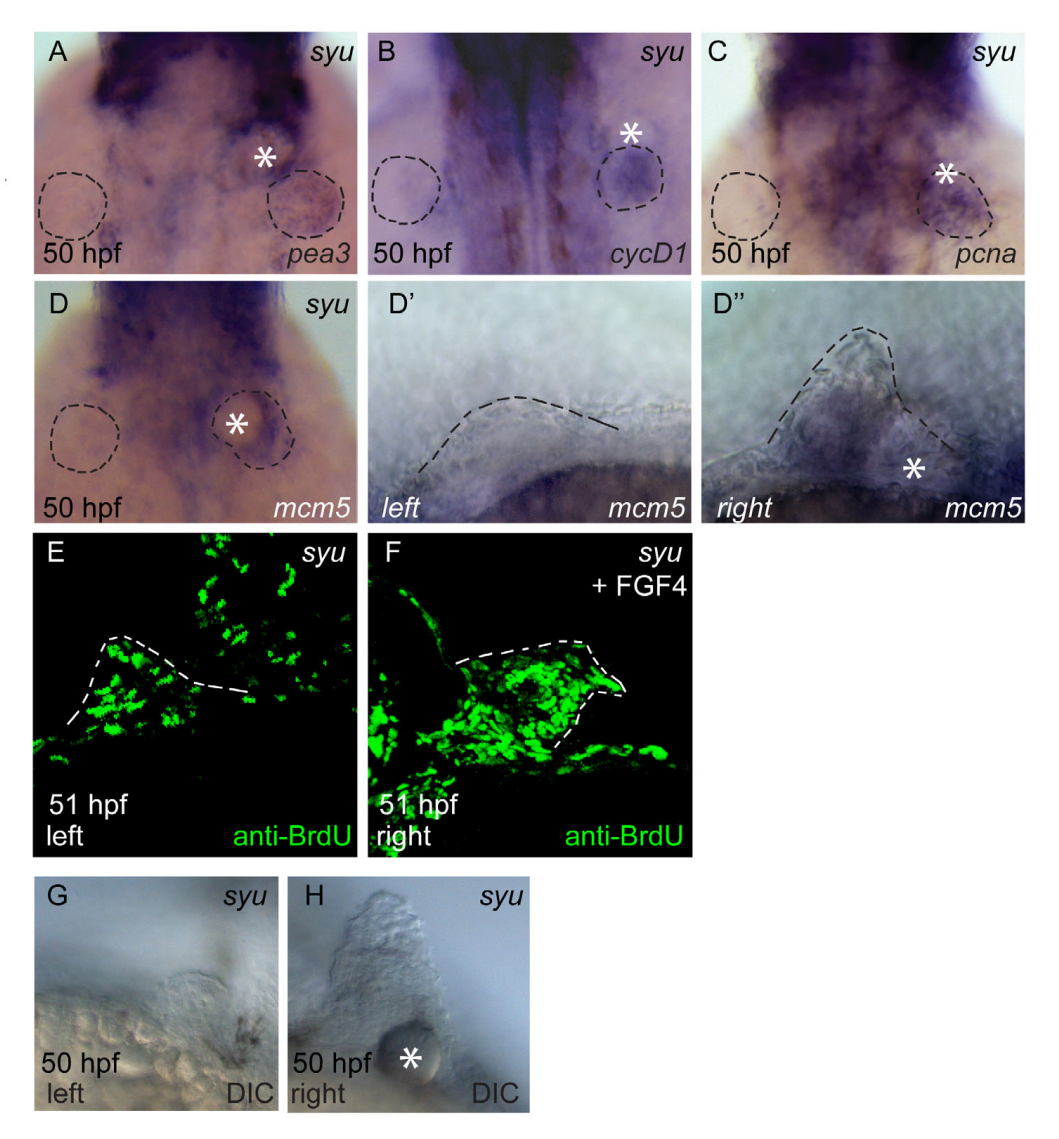
**Implantation of FGF4-soaked beads rescues G1- and S-phase cell-cycle gene expression and S-phase progression in *sonic-you *fin buds**. Fin buds on the right hand side of *sonic-you *embryos were implanted with FGF4-soaked heparin beads at 29–32 hpf, grown until 50 hpf and fixed (A-D, F-H). For anti-BrdU staining, embryos were first implanted and then injected with 10 mM BrdU solution at 38 hpf before fixation at 50 hpf. *Sonic-you *embryos show upregulation of *pea3 *(A), *cyclinD1 *(B), *pcna *(C) and *mcm5 *(D) expression in response to the FGF4-soaked beads on the implanted side. Fin buds are outlined by dotted lines in panels A to D. A non-implanted fin bud on the left hand side shows no *mcm5 *expression (D'), while an implanted fin bud on the right hand side of the same embryo (D") shows restored *mcm5 *expression. A non-implanted fin bud shows few BrdU-labeled nuclei (E), while an FGF4 bead-implanted fin bud (F) has extensive BrdU labeling (sections of both sides of 10 bead-implanted embryos were analysed). Fin buds implanted with FGF4 beads show increased outgrowth (D", F, H), compared to non-implanted control fin buds (D', E, G).

The results presented so far strongly suggest that Fgf signaling is directly required for cell-cycle progression in zebrafish fin buds, while Shh plays an indirect role via its regulation of Fgf expression. However, since the Shh and Fgf signaling pathways in the limb bud are coupled by a feedback loop mechanism, it is difficult to change the activity of one pathway without affecting the other. Therefore, we decided to use a gain-of-function experiment to uncouple the Fgf pathway completely from the Shh pathway, by providing an ectopic source of Fgf protein in *shh *mutant fin buds, and asking if this ectopic source of Fgf signaling is able to rescue cell-cycle progression in the absence of Shh. For this purpose, heparin gel beads were soaked with recombinant human FGF4 protein and implanted into fin buds on the right hand side of *shh *mutant embryos at 29–32 hpf. The fin buds on the left hand side were not implanted and served as an internal control in these experiments. Implanted embryos were then grown to 50 hpf and gene expression was analysed by *in situ *hybridisation. We find that FGF4-soaked beads induce *pea3 *expression in *shh *mutant fin buds (Fig. 5A). Furthermore, *cyclinD1 *(Fig. 5B), *pcna *(Fig. 5C) and *mcm5 *(Fig. 5D, D', D") transcripts are also induced in fin buds implanted with FGF4-soaked beads. Consistent with these results, we also detect increased incorporation of BrdU in bead-implanted *shh *mutant fin buds, compared to unimplanted fin buds (Fig. 5E, F). Finally, we also observe increased growth of *shh *mutant fin buds with implanted FGF4-soaked beads (Fig. 5D', D", F, G), further supporting the conclusion that Fgf signaling is able to restore outgrowth in the absence of Shh. This increase in fin size after bead-implantation is somewhat variable and depends on bead position relative to the fin bud, with the largest outgrowth observed when beads are located right inside the bud (Fig 5F, G). Taken together, these results indicate that Fgf signaling is sufficient to direct proliferation in zebrafish fin buds in the absence of Shh.

## Discussion

The cell-cell signaling events that direct vertebrate limb development have been the subject of intense research for more than a hundred years. This provides an excellent foundation for investigating the mechanisms whereby pattern formation is integrated with proliferation. In this study we have focused on two of the main signals important for patterning and growth of the vertebrate limb: the Shh and Fgf signaling pathways. While both signals are crucial for outgrowth of the limb bud, it has been very challenging to uncouple these signals from each other, since expression of Shh depends on Fgf signaling, and vice versa. For example, while AER ablation experiments have been interpreted as causing failure of limb outgrowth because they remove the source of Fgf signaling from the limb bud [10,11,43], AER ablation simultaneously leads to loss of Shh expression from the ZPA [13,14], and so cannot be used to separate the effect of Fgf on proliferation from the effect of Shh. To overcome this challenge, we have used a combination of loss-of-function and gain-of-function experiments in the the zebrafish model system to uncouple Shh from Fgf signaling in the pectoral fin bud, and have assessed the effect of each signal on fin bud proliferation independently of the other signal.

Our results show that the effect of Shh on proliferation during limb development is indirect, and is mediated by its effect on Fgf expression in the AER. Inhibition of Shh signaling leads to loss of cell-cycle progression only after a relatively long delay period of around 13 hours, and this correlates with a concomitant loss of Fgf signaling. Inhibition of Fgf signaling, on the other hand, leads to loss of cell-cycle progression very rapidly, after only 3 hours of inhibitor treatment, and this occurs even though activity of the Shh pathway is still present. This rapid effect of Fgf on cell-cycle progression suggests a direct transcriptional response of cell-cycle genes to the Fgf pathway in the limb bud, which is consistent with the direct mitogenic effect of Fgf signaling shown on several cell types in tissue culture.

Since Hh signaling has also been shown to have a direct mitogenic effect on some cell types, it is perhaps surprising that Shh directs proliferation indirectly in the vertebrate limb bud. However, there is at least one previous example of such an indirect effect of Hh signaling on proliferation. During *Drosophila *wing development, Hh is necessary for growth of the wing imaginal disc, but this effect is mediated via the Hh-dependent expression of Decapentaplegic (Dpp), a member of the Tgf-β family of secreted signaling proteins [44-46]. Furthermore, the proliferative response of different cell types to Hh is clearly context-dependent, and Hh signaling can even function as a negative regulator of the cell-cycle in some cell types [47]. Interestingly, this negative effect of Hh signaling on proliferation also appears to be indirect in some cases. In the rodent colonic epithelium, for example, Hh signaling stimulates cell-cycle exit by antagonizing the Wnt pathway [48]. In the *Drosophila *retina, on the other hand, Hh signaling has both positive and negative effects on cell-cycle progression [49]. Cell-cycle arrest of cells in front of the retinal differentiation wave depends on Hh signaling in combination with Dpp, while cell-cycle re-entry behind the wave front also depends on Hh signaling, but in this case mediated by the Notch signaling pathway [49,50].

Our results also show a context-dependent effect of Fgf signaling on cell-cycle progression. Thus Fgf signaling is clearly essential for cell-cycle progression in the pectoral fin buds and in the branchial arches, since expression of G1- and S-phase cell-cycle genes in these tissues is lost after only 3 hours of inhibition of the Fgf pathway. Inhibition of Fgf signaling fails to affect cell-cycle progression in other organs, however, such as the retina and the optic tectum, or at earlier stages of development. The Fgf signaling pathway is therefore not a global mitogenic signal in the zebrafish embryo, but instead directs proliferation in a highly tissue-specific manner. Altogether, the evidence thus indicates that both the Hh and Fgf pathway affect cell-cycle progression in some cell types but not in others, and that this effect can be either direct or indirect. The control of cell-proliferation in multicellular organisms can therefore only be understood in a context-dependent manner, and our results help to shed light on this question in the context of the vertebrate limb bud. The molecular mechanisms by which different cell types respond distinctly to the same signal are still poorly understood, but will undoubtedly be unravelled by future research.

## Conclusion

Our data show that Shh and Fgf signaling have distinct effects on proliferation during vertebrate limb development. While both genes are necessary for outgrowth of the limb bud, the role of Shh in this process is indirect, and is mediated via its effect on expression of Fgf genes in the AER. In contrast, the effect of Fgf signaling on cell-cycle progression in the limb bud appears to be direct, and Fgf signaling is both necessary and sufficient to direct proliferation, even in the absence of Shh. Together with the work of others, our results indicate that the role of both signaling pathways in regulating proliferation is highly context dependent, and our results shed light on their function in directing proliferation in the context of limb development.

## Methods

### Fish stocks, maintenance and care

Wild-type *Tupfel Long Fin (TLF) *and *sonic-you*^t4 ^heterozygous fish were used. Fish were maintained according to standard protocols. Embryos were grown in E3 embryo medium at 28°C with or without the addition of 0.003% 1-phenyl-2-thiourea (PTU, Sigma) to inhibit pigmentation. Staging was performed according to hours post-fertilization (hpf) [51].

### Chemical inhibitors and treatment procedures

FGF signaling inhibitor SU5402 (Calbiochem, cat # 572630) was dissolved in DMSO at 8 mM. Treatment was performed with a 10 μM solution of SU5402 or a corresponding control DMSO solution in E3 embryo medium. Cyclopamine (Toronto Research Chemicals, cat# C988400) was dissolved in ethanol at 20 mM. Treatment was performed with 100 μM solution of cyclopamine or control ethanol solution in E3 embryo medium.

### In situ hybridisation and probe synthesis

RNA in situ hybridisations were performed according to Jowett [52]. Probes were made using Roche DIG RNA Labeling Kit (Cat # 11175025910). cDNAs used to make antisense probes: *ptc1*, *pea3 *[53], *cyclin D1 *[54], *pcna *(cDNA was ampplified using pcna_for: CCTACTCCAAACTAAGAAAGCAGCA and pcna_rev: ATCGGGAATCCATTGAACTGG), *mcm5 *(cDNA was amplified using mcm5_for: TGGTGGAGGAGAAAGCGTCG and mcm5_rev: GGCCTCATGGATTGCGACTC and cloned into pGEM-T-easy (Promega)), *ra1 *(NM199811) cDNA was ampplified using ra1_for: CCATTGGAGGAAACAGGAGA and T7_ra1_rev: TAATACGACTCACTATAGGGcagcgtgtacctggtaagca and transcribed from the PCR product using T7 polymerase).

### Antibody staining

Antibody stainings were performed on 14 μm cryosections. Cryosections were rehydrated in PBS with 0.1% Tween-20 (PBST), treated for 20 min with 4N HCl, washed several times in PBST and blocked for 1 hour with blocking solution (4% normal goat serum in PBST). Mouse anti-BrdU antibody (1:100) (Roche, cat# 1 170 376) and secondary anti-mouse Alexa Fluor 488 antibody (1:400) (Invitrogen, cat# A11001) were diluted in blocking solution and incubated with the sections for 2 hours each. Images were taken using Leica SP2 confocal microscope and processed using Adobe Creative Suite CS2.

### Bead implantation

Bead implantation was performed as described before [53]. Recombinant human Fgf4 protein (R&D Systems, cat# 235-F4-025) was dissolved at a concentration 250 ng/μl in PBS with 0.1% bovine serum albumin and mixed in proportion 1:1 with the mix of beads filtered through a 70 μm Cell strainer (BD Falcon, cat# 352350).

## Authors' contributions

CJN suggested the idea of checking the relative importance of Shh and FGF signaling pathways in driving proliferation in zebrafish fin buds, guided the project and helped drafting the manuscript. SVP designed and performed experiments and drafted the manuscript. All authors read and approved the final manuscript.
